# Draft Genome and Comparative Analysis of a *Cutaneotrichosporon jirovecii*-Related Yeast Recovered from a Human Fecal Sample

**DOI:** 10.3390/jof12060450

**Published:** 2026-06-20

**Authors:** Yuyan Huang, Rongchen Dai, Feiyi Liu, Xiaoyan Gou, Renyuan Zhu, Shuying Yu, Zhengyu Luo, Dan Guo, Tianshu Sun, Meng Xiao, Yingchun Xu, Lina Guo

**Affiliations:** 1 Department of Laboratory Medicine, State Key Laboratory of Complex, Severe and Rare Diseases, Peking Union Medical College Hospital, Chinese Academy of Medical Sciences and Peking Union Medical College, Beijing 100730, China; b2023001071@pumc.edu.cn (Y.H.); chewxway@163.com (R.D.); lfy199601100480610@163.com (F.L.); gxiaoyan1999@163.com (X.G.); zhury1978@163.com (R.Z.); yushuying2016@163.com (S.Y.); luozhengyujy@163.com (Z.L.); sun_tianshu@163.com (T.S.); cjtcxiaomeng@aliyun.com (M.X.); 2 Graduate School, Chinese Academy of Medical Sciences and Peking Union Medical College, Beijing 100730, China; 3 Center for Biomedical Technology, National Infrastructures for Translational Medicine, Institute of Clinical Medicine, Peking Union Medical College Hospital, Chinese Academy of Medical Sciences and Peking Union Medical College, Beijing 100730, China; gdp1225@163.com; 4 State Key Laboratory of Complex, Severe, and Rare Diseases, Chinese Academy of Medical Sciences and Peking Union Medical College, Beijing 100730, China; 5 Clinical Biobank, Peking Union Medical College Hospital, Chinese Academy of Medical Sciences and Peking Union Medical College, Beijing 100730, China

**Keywords:** *Cutaneotrichosporon jirovecii*, Trichosporonaceae, draft genome, average nucleotide identity, ITS phylogeny, gut mycobiome, CAZyme

## Abstract

Background: *Cutaneotrichosporon jirovecii* is an under-characterized basidiomycetous yeast within the family Trichosporonaceae. Its taxonomic placement, ecological distribution, and functional potential remain incompletely understood because genome-scale resources for *C. jirovecii* and closely related lineages are limited. Methods: We characterized strain H0426_7, a *C. jirovecii*-related yeast recovered from a human fecal sample, using ITS-based type-strain comparison, ITS phylogenetic analysis, whole-genome sequencing, average nucleotide identity analysis, read-level assessment of public *C. jirovecii*-labeled datasets, and comparative functional annotation. Antifungal susceptibility was assessed using the Sensititre YeastOne plate. Results: The ITS sequence of H0426_7 closely matched type-strain material of *C. jirovecii*, including CBS 6864 and its equivalent deposits. The ITS-based tree placed H0426_7 adjacent to CBS 6864 with bootstrap support of 87%. The final draft genome comprised 38.66 Mb in 1974 contigs, with a GC content of 63.76% and BUSCO completeness of 80.0%. ANI analysis showed that H0426_7 was genomically distinct from the recognized *Cutaneotrichosporon* species included in the ANI analysis but highly similar to two unclassified feces-derived strains, P10-008 and PK4640, with ANI values exceeding 98.8%. Two public datasets labeled as *C. jirovecii* showed anomalously low ANI values with H0426_7; read-level taxonomic profiling indicated low target-fungal read proportions, suggesting that these datasets are unsuitable as definitive genome-level references. CAZyme annotation identified 285 family assignments in H0426_7, representing 278 non-redundant predicted proteins, including relatively high GH5 and GH31 counts, suggesting candidate carbohydrate-utilization features shared with the H0426_7/P10-008/PK4640 lineage. Conclusions: H0426_7 is best described as a *C. jirovecii*-related *Cutaneotrichosporon* isolate pending availability of a high-quality genome assembly from the *C. jirovecii* type strain. This study expands genome-scale resources for underrepresented basidiomycetous yeasts and provides a comparative framework for future taxonomic, ecological, and functional studies of feces-associated *Cutaneotrichosporon* lineages.

## 1. Introduction

Species of the genus *Cutaneotrichosporon* are basidiomycetous yeasts formerly accommodated within the broader *Trichosporon*-like group. Members of this lineage have been recovered from diverse environmental and host-associated niches, including animals and humans [1,2,3]. Although many species in this group have historically been regarded as environmental yeasts, members of the family Trichosporonaceae are increasingly recognized in clinical microbiology, either as colonizers or as opportunistic pathogens in immunocompromised hosts [4]. The taxonomy of *Trichosporon*-like yeasts has undergone substantial revision following multilocus and phylogenomic studies, leading to the recognition of genera such as *Cutaneotrichosporon*, *Apiotrichum*, and related taxa [5]. These revisions have improved the systematic framework of the group, but they have also exposed persistent challenges in species delimitation, particularly for rare taxa represented by sparse reference sequences and limited genome resources.

*Cutaneotrichosporon jirovecii* is a validly described species within this revised taxonomic framework. Its identification has relied largely on phenotypic characteristics and ribosomal DNA markers [6,7]. However, genome-scale information for *C. jirovecii* and closely related lineages remains scarce. This lack of genomic data constrains accurate species assignment, comparative genomic analysis, and assessment of the ecological and functional characteristics of isolates related to the *C. jirovecii* type strain. For rare basidiomycetous yeasts, single-gene barcoding may be insufficient to resolve closely related lineages, particularly when public databases contain few type-strain-derived records or assemblies of uneven quality. Genome-resolved analysis is therefore essential for clarifying the taxonomic position and biological potential of underrepresented *Cutaneotrichosporon* lineages.

The clinical relevance of *C. jirovecii* may be underestimated. In routine diagnostic practice, uncommon *Trichosporon*/*Cutaneotrichosporon* isolates are frequently difficult to identify accurately at the species level using phenotypic methods or matrix-assisted laser desorption ionization-time of flight mass spectrometry (MALDI-TOF MS) when reference spectra or molecular reference data are incomplete [3,4,7]. This diagnostic limitation is relevant because published reports have described *C. jirovecii* or closely related taxa in host-associated contexts, including oral colonization, human superficial or urinary samples, and animal infections [2,3,7,8,9,10,11,12]. These observations do not establish that *C. jirovecii* is a common human pathogen, but they indicate that this species has broader clinical and host-associated relevance than is currently reflected in available genomic resources.

The human gut mycobiome contains a diverse but unevenly characterized fungal community. Large-scale studies of healthy individuals have shown that gut-associated fungi include both ascomycetous and basidiomycetous yeasts, although fungal abundance and composition are more variable than bacterial communities [13]. Much of the gut mycobiome literature has focused on *Candida* and other ascomycetous yeasts, whereas basidiomycetous yeasts remain less systematically characterized. This imbalance partly reflects their lower abundance, intermittent recovery, and limited representation in reference databases. Consequently, rare or poorly represented basidiomycetous yeasts may be overlooked or misclassified in both culture-dependent and culture-independent studies.

The ecological interpretation of a yeast recovered from feces requires caution. A fecal isolate may represent a resident gut organism, transient passage after environmental or dietary exposure, or temporary enrichment under altered ecological conditions. This distinction is particularly relevant in *Clostridioides difficile* infection (CDI), which is characterized by profound disruption of the intestinal bacterial community and altered gut metabolic conditions [14]. Although bacterial dysbiosis in CDI has been extensively studied, the fungal component of CDI-associated microbiota remains less well defined [15]. Detection of uncommon yeasts in CDI-associated fecal specimens should therefore be interpreted as an ecological observation rather than evidence of causation. Nevertheless, genome-resolved characterization of such isolates can provide useful reference data for future studies of fungal diversity in disturbed intestinal ecosystems.

Beyond taxonomy and clinical identification, *C. jirovecii* and related taxa are of interest because members of Trichosporonaceae display diverse metabolic capabilities, including broad carbon utilization, enzyme production, and adaptation to heterogeneous environments [16,17,18]. Comparative analysis of carbohydrate-active enzymes (CAZyme), transporter systems, and other functional gene repertoires may help generate hypotheses regarding substrate utilization and ecological fitness. Such analysis is especially important for poorly characterized lineages, where genome data remain sparse and species boundaries are incompletely resolved.

In this study, we report the draft genome sequence and comparative genomic characterization of strain H0426_7, a *C. jirovecii*-related yeast isolated from a fecal sample of a *C. difficile* toxin-positive patient. We combined internal transcribed spacer (ITS)-based type-strain comparison, ITS phylogenetic analysis, average nucleotide identity (ANI) analysis, read-level assessment of public *C. jirovecii*-labeled datasets, genome annotation, and comparative CAZyme profiling. Our aim was to clarify the phylogenetic placement of H0426_7 within *Cutaneotrichosporon*, provide a genome-scale resource for an underrepresented lineage, and generate cautious hypotheses regarding its metabolic potential and feces-associated occurrence without inferring a causal relationship with CDI.

## 2. Materials and Methods

### 2.1. Strain Isolation and DNA Extraction

Strain H0426_7 was isolated from a human fecal sample collected in Beijing, China, in 2023. The fecal sample was inoculated onto Sabouraud dextrose agar (SDA) and incubated at 35 °C. After 48 h of incubation, H0426_7 was observed as the only yeast-like colony morphotype on the primary isolation plate, with the semi-quantitative growth density recorded as “+”. No additional fungal colony morphotypes were observed. The isolate was purified by subculturing onto fresh SDA plates.

Genomic DNA was extracted from a pure culture using a bacterial/fungal DNA extraction kit (Majorbio, Shanghai, China) according to the manufacturer’s instructions. Purified genomic DNA was quantified, and high-quality DNA was used for subsequent analyses.

### 2.2. Antifungal Susceptibility Testing

Antifungal susceptibility testing was performed using Sensititre YeastOne plates (Thermo Fisher Scientific) following the manufacturer’s instructions [19]. The panel contained nine antifungal agents: amphotericin B (0.12–8 µg/mL), 5-flucytosine (0.06–64 µg/mL), anidulafungin (0.015–8 µg/mL), caspofungin (0.008–8 µg/mL), micafungin (0.008–8 µg/mL), fluconazole (0.12–256 µg/mL), itraconazole (0.015–16 µg/mL), posaconazole (0.008–8 µg/mL), and voriconazole (0.008–8 µg/mL).

Fungal growth was indicated by a color change from blue to red or purple, whereas inhibition was indicated by the absence of color change. The minimum inhibitory concentration (MIC) was recorded as the lowest antifungal concentration showing inhibition of color change relative to the positive growth control.

Because species-specific clinical breakpoints and epidemiological cutoff values have not been established for *Cutaneotrichosporon jirovecii* or closely related *Cutaneotrichosporon* taxa, MIC values were interpreted descriptively rather than assigned to clinical susceptibility categories. CLSI M27, 4th edition, was used as the reference standard for general yeast antifungal susceptibility testing principles and interpretive context [20].

### 2.3. ITS and IGS Sequencing for Species Identification

Preliminary species identification was performed based on sequencing of the ITS region and the intergenic spacer 1 (IGS1) region. The ITS region was amplified using primers ITS1 (5′-TCCGTAGGTGAACCTGCGG-3′) and ITS4 (5′-TCCTCCGCTTATTGATATGC-3′). The IGS1 region was amplified using primers IGS1-F (5′-ATCCTTTGCAGACGACTTGA-3′) and IGS1-R (5′-AGCTTGACTTCGCAGATCGG-3′). PCR products were subjected to Sanger sequencing.

Consensus sequences were generated from forward and reverse reads and queried against the NCBI nucleotide collection using BLASTn (https://blast.ncbi.nlm.nih.gov/Blast.cgi, accessed on 31 May 2026). For ITS-based species-affinity assessment, BLAST searches were restricted to sequences derived from type material where applicable, with particular attention to the type strain *C. jirovecii* CBS 6864 and its equivalent deposits. The IGS1 sequence was examined as auxiliary information but was not used as the primary basis for type-strain-level assignment because no informative *C. jirovecii* type-material match with adequate coverage was obtained under the current database coverage.

### 2.4. Library Construction and Sequencing

Genomic DNA was fragmented into approximately 400 bp fragments using a Covaris M220 Focused-ultrasonicator (Covaris, LLC, Woburn, MA, USA) according to the manufacturer’s protocol. Illumina sequencing libraries were prepared using the NEXTFLEX Rapid DNA-Seq Kit (Revvity, Waltham, MA, USA). Briefly, DNA fragments were end-repaired, A-tailed, and ligated to sequencing adapters, followed by PCR amplification to enrich adapter-ligated fragments.

The prepared libraries were sequenced on the Illumina NovaSeq 6000 platform (Illumina Inc., San Diego, CA, USA) using a paired-end configuration of 151 cycles from each end. Raw sequencing generated approximately 2 Gb of raw sequence data. Raw reads were quality-filtered using fastp v0.23.2 to remove adapter sequences, low-quality reads, reads containing excessive ambiguous bases, and reads that were too short. After filtering, high-quality clean reads with a mean read length of approximately 150 bp from each end were retained for subsequent *de novo* genome assembly.

### 2.5. Genome Assembly, Contig Filtering, and Annotation

High-quality clean reads were assembled *de novo* using SPAdes v3.19.1. To reduce potential contamination and sequence redundancy, assembled contigs were subjected to a reference-guided filtering procedure. Briefly, contigs were aligned using BLASTn against a genus-level reference database consisting of 36 publicly available *Cutaneotrichosporon* genome assemblies downloaded from NCBI (Supplementary Table S5), with an E-value threshold of 1 × 10^−5^. Contigs without significant matches to this reference database were considered potential non-target sequences and excluded from the final assembly. The remaining contigs were further clustered using CD-HIT v4.8.1 with the parameters -G 0 -aS 0.9 -g 1 -c 0.99 -l 500, and contigs shorter than 1000 bp were removed. The resulting non-redundant contig set was used as the final draft genome assembly.

Genome completeness was assessed using BUSCO v3.0.2 with the fungi_odb9 lineage dataset. Protein-coding genes were predicted using Augustus v2.7 with the *Ustilago maydis* training model, and rRNA genes were identified using Barrnap v0.8. Functional annotation was performed by comparison against public databases, including the NCBI non-redundant protein database, Swiss-Prot, Gene Ontology, KEGG, Pfam, and KOG. Gene Ontology annotation was assigned using InterProScan v5.77-108.0, and KEGG annotation was performed using KAAS.

Carbohydrate-active enzymes were annotated separately using dbCAN2 against the HMMdb and CAZy databases. CAZyme family assignments were summarized independently from the general functional annotation. Transporter-associated genes were identified by BLASTp searches against the Transporter Classification Database. Genes potentially related to host interaction, pathogenicity, or fungal virulence were screened against PHI-base and the Database of Fungal Virulence Factors using BLASTp, with thresholds of E-value ≤ 1 × 10^−5^, identity ≥ 30%, and coverage ≥ 50%.

### 2.6. Comparative Genomic Analysis and Average Nucleotide Identity

ANI analysis was performed using FastANI v1.33 to assess genome-wide relatedness between strain H0426_7 and representative publicly available *Cutaneotrichosporon* genomes [21]. Pairwise ANI values were calculated between H0426_7 and selected *Cutaneotrichosporon* genomes, including recognized species, the unclassified strains *Cutaneotrichosporon* sp. P10-008 and PK4640, and two public datasets labeled as *C. jirovecii* (ERR2799408 and ERR3040836). FastANI was run with default parameters. ANI values were interpreted with reference to the commonly used 95% genome-level reference threshold originally proposed for prokaryotic species delineation and subsequently applied in comparative genomic studies of fungi and yeasts [22].

For the two public datasets labeled as *C. jirovecii*, read-level taxonomic composition reported in the NCBI SRA was examined to evaluate their suitability for genome-level comparative analysis.

CAZyme repertoires of H0426_7 and representative *Cutaneotrichosporon* genomes were compared using the same dbCAN2-based annotation pipeline. Class-level distributions and selected family-level assignments, including GH5 and GH31, were summarized for comparative analysis.

### 2.7. ITS-Based Phylogenetic Analysis

ITS sequences of H0426_7 and representative *Cutaneotrichosporon* taxa were included in the phylogenetic analysis. Reference ITS sequences were downloaded from the CBS database, the National Genomics Data Center, and NCBI, prioritizing type or reference strains where available. Genome-derived ITS sequences were extracted from the assembled genomes of *Cutaneotrichosporon* sp. P10-008 and PK4640.

Sequences were aligned using MAFFT v7.450. The resulting alignment was manually inspected, and terminal overhangs or poorly aligned terminal regions were trimmed in BioEdit. The final trimmed alignment was used to construct a neighbor-joining phylogenetic tree in MEGA X. Evolutionary distances were computed using the Maximum Composite Likelihood method, and branch support was assessed using 1000 bootstrap replicates. Bootstrap support values were shown on the tree. Both the original tree and the bootstrap consensus tree were exported for visualization.

## 3. Results

### 3.1. Literature Review of Cutaneotrichosporon jirovecii

*Cutaneotrichosporon jirovecii* belongs to the basidiomycetous yeast family Trichosporonaceae. In earlier literature, the species was reported as *Trichosporon jirovecii*. Following the integrated phylogenetic reclassification of Tremellomycetes, several former *Trichosporon*-like taxa were redistributed into revised genera, including *Cutaneotrichosporon*, *Apiotrichum*, and related lineages [5]. This taxonomic history is important because older clinical, ecological, and applied microbiology studies may still use the former combination *T. jirovecii*, whereas current taxonomy places the species within *Cutaneotrichosporon*.

Published reports concerning *C. jirovecii*/*T. jirovecii* remain relatively sparse and heterogeneous. Human-associated studies have mainly appeared in broader surveys of oral yeasts, superficial infection-associated Trichosporonales, urinary isolates, and medically relevant Trichosporonaceae [2,3,4,7]. In these studies, *C. jirovecii* has not been recognized as a commonly reported human pathogen. Rather, it appeared as one of several uncommon Trichosporonaceae taxa requiring molecular confirmation for reliable species-level identification. This is consistent with previous work showing that rare *Trichosporon*, *Apiotrichum*, and *Cutaneotrichosporon* species are difficult to resolve by phenotypic approaches alone and often require sequencing-based identification [4,6,7].

Animal-associated reports further broaden the known host range of *T. jirovecii*. The species has been reported in a respiratory tract infection in a dog, cutaneous lesions in a tortoise, and disease-associated isolation from red swamp crayfish [8,11,12]. These reports suggest that *T. jirovecii* can be recovered from diverse host-associated contexts, including mammals, reptiles, and crustaceans. However, the current evidence remains case-based or small-scale, and it does not establish host specificity, prevalence, or a defined pathogenic role. More broadly, studies of *Trichosporon* isolates from animal hosts also illustrate the ecological breadth and identification complexity of Trichosporonaceae [9].

In addition to clinical and host-associated reports, *T. jirovecii* has appeared in applied microbiology studies. It has been used in L-cysteine-desulfhydrase-based biosensing and in yeast-mediated cadmium sulfide nanoparticle synthesis [17,18]. These studies indicate cultivability and metabolic potential, but they were not designed to resolve species boundaries, clinical relevance, or genome-scale functional characteristics. At the family level, comparative genomic work on Trichosporonaceae has shown broad metabolic diversity and lifestyle variation, but *C. jirovecii* itself remains poorly represented in genome-resolved studies, limiting understanding of its evolutionary relationships, functional potential, and ecological distribution [16].

Overall, the published literature indicates that *C. jirovecii* is a taxonomically valid but under-characterized basidiomycetous yeast, with scattered reports across human-associated, animal-associated, and applied microbiology contexts. Existing studies remain largely case-based, survey-based, or application-oriented, and genome-resolved information on this species remains limited compared with better-characterized members of Trichosporonaceae, providing the rationale for the genome-based characterization and comparative analyses presented in this study.

### 3.2. Clinical Characteristics and Antifungal Susceptibility Profiles

Strain H0426_7 was isolated from a fecal sample obtained from a 69-year-old male patient admitted to the Department of Gastroenterology because of diarrhea. The patient tested positive for *C. difficile* toxin, and the stool sample was described as yellow and soft. On Sabouraud dextrose agar (SDA), H0426_7 was the only yeast-like colony morphotype recovered from the stool sample, with the semi-quantitative growth density recorded as “+”. No additional fungal colony morphotypes were observed on the primary isolation plate. The isolate showed low MIC values against triazoles and amphotericin B: voriconazole (0.015 µg/mL), posaconazole (0.06 µg/mL), itraconazole (0.06 µg/mL), fluconazole (0.5 µg/mL), and amphotericin B (0.25 µg/mL). 5-Flucytosine also showed a relatively low MIC value (2 µg/mL). The MIC values for echinocandins were caspofungin (0.25 µg/mL), micafungin (0.5 µg/mL) and anidulafungin (0.25 µg/mL). Detailed MIC values are summarized in Table 1.

Because standardized clinical breakpoints or epidemiological cutoff values have not been established for *Cutaneotrichosporon*, these MIC data were interpreted descriptively rather than as categorical susceptible or resistant results. Overall, the antifungal susceptibility profile of H0426_7 was characterized by low MIC values for triazoles, amphotericin B, echinocandins, and 5-flucytosine, a pattern broadly consistent with previously reported echinocandin susceptibility reported in several members of Trichosporonaceae.

### 3.3. Strain Identification Based Primarily on ITS Sequence Analysis

Initial molecular identification was performed using the ITS region. The ITS sequence of strain H0426_7 was 530 bp in length. BLAST analysis restricted to sequences derived from type material showed that the ITS sequence of H0426_7 closely matched type-strain material of *C. jirovecii*. The best matches included *Trichosporon jirovecii* strain ATCC 34499, an equivalent deposit of the *C. jirovecii* type strain, with 100% query coverage and 99.62% identity (accession HM802131.1), and *C. jirovecii* CBS 6864, with 100% query coverage and 99.62% identity (accession NR_073252.1). An additional CBS 6864-derived ribosomal sequence also showed high similarity, with 98% query coverage and 99.61% identity (Table S1).

The IGS1 sequence was also queried against type-material records; however, no informative *C. jirovecii* type-material match with adequate coverage was obtained under the current database.

### 3.4. Genome Sequencing and Assembly Statistics

Whole-genome sequencing of strain H0426_7 was performed using the Illumina platform. After quality filtering with fastp, 13,884,374 clean reads were retained, corresponding to 2.09 Gb of clean data and an estimated average sequencing depth of approximately 54×. *De novo* assembly of the high-quality reads yielded a draft genome comprising 1974 contigs, with a total length of 38,658,432 bp and an overall GC content of 63.76%. The longest contig was 201,186 bp, and the contig N50 was 30,278 bp. The overall GC content of 63.76% was comparable to values reported for other members of the genus *Cutaneotrichosporon*.

Genome completeness was assessed using BUSCO v3.0.2 with the fungal lineage dataset. The assembly contained 80.0% complete BUSCOs, including 37.0% single-copy and 43.0% duplicated BUSCOs; 1.7% of BUSCOs were fragmented and 18.3% were missing (Table 2). Before post-assembly filtering, the initial assembly showed 86.4% complete BUSCOs with 54.3% duplicated BUSCOs. After reference-guided contaminant screening, CD-HIT redundancy removal, and length filtering, the final assembly retained fewer duplicated BUSCOs while maintaining 80.0% complete BUSCOs.

### 3.5. Genome Annotation and Functional Profiling

**Figure 1:**
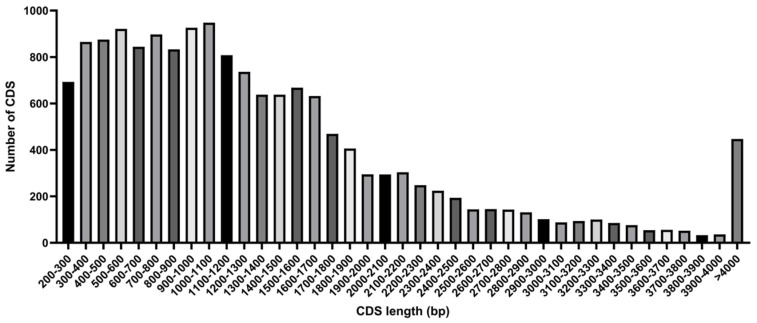
Distribution of predicted coding sequence lengths in strain H0426_7. The x-axis represents coding sequence length in base pairs, and the y-axis represents the number of coding sequences within each 100 bp interval. More than 70% of predicted coding sequences were distributed within the 200–1500 bp interval.

Genome annotation identified 16,142 predicted protein-coding genes in strain H0426_7, with an average gene length of 1382 bp. Coding sequences accounted for approximately 57.69% of the assembled genome. The length distribution of coding sequences is shown in Figure 1.

Homology-based searches showed that 12,024 genes (74.5%) had significant matches in the NR database, whereas 3611 genes (22.4%) were annotated in the Swiss-Prot database. Protein domain analysis using Pfam and orthologous group classification using KOG were further performed to characterize the predicted gene set.

Gene Ontology (GO) annotation assigned at least one GO term to 6030 predicted genes, covering the Biological Process, Molecular Function, and Cellular Component categories. Major annotated terms included transmembrane transport and metabolic process under Biological Process, protein binding and oxidoreductase activity under Molecular Function, and integral component of membrane and nucleus under Cellular Component. KEGG pathway analysis identified 2725 genes involved in known metabolic and cellular pathways, including carbohydrate metabolism, amino acid metabolism, and energy metabolism. Representative enzymes included alcohol dehydrogenase, aldehyde dehydrogenase, and chitin synthase. Detailed annotation results are provided in Supplementary Table S2.

CAZyme annotation yielded 285 family assignments in H0426_7, representing 278 non-redundant predicted proteins. These assignments covered glycoside hydrolases, glycosyltransferases, polysaccharide lyases, carbohydrate esterases, auxiliary activity enzymes, and carbohydrate-binding modules. To provide comparative genomic context, CAZyme repertoires were compared between H0426_7 and 14 publicly available *Cutaneotrichosporon* genomes using the same annotation pipeline. These public genomes included the unclassified strains *Cutaneotrichosporon* sp. P10-008 and PK4640, recognized *Cutaneotrichosporon* species, and two public datasets labeled as *C. jirovecii* (ERR2799408 and ERR3040836). According to public BioSample metadata, P10-008 and PK4640 were both isolated in 2019 from fecal samples of healthy human donors in Dalian, China. Because read-level taxonomic profiling indicated low target-fungal read proportions in ERR2799408 and ERR3040836 (see Section 3.6), these two datasets were used only as exploratory references in the functional comparison. The distribution of CAZyme classes is summarized in Table 3, and selected family-level results for GH5 and GH31 are provided in Supplementary Table S3.

At the family level, H0426_7 contained 16 GH5 assignments and 7 GH31 assignments. GH5 includes enzymes involved in the degradation of cellulose, hemicellulose, and other plant cell wall polysaccharides, whereas GH31 includes α-glucosidases involved in the hydrolysis of starch-derived α-linked glucans. The closely related strains P10-008 and PK4640, both derived from healthy human feces, also showed high GH5 counts, with 18–19 assignments, and both contained 7 GH31 assignments. This pattern suggests that selected carbohydrate-degrading enzyme families may represent a candidate genomic feature of this feces-derived H0426_7/P10-008/PK4640 lineage. Given the limited number of available strains and the draft nature of the genomes, this observation should be considered hypothesis-generating rather than evidence of a defined gut-adaptation trait.

The two public datasets labeled as *C. jirovecii* contained fewer CAZyme family assignments than H0426_7, P10-008, and PK4640. However, because these datasets showed low target-fungal read proportions and evidence of non-target reads, their reduced CAZyme counts may reflect incomplete or contaminated assemblies rather than true biological reduction. Therefore, CAZyme comparisons involving ERR2799408 and ERR3040836 were not used to infer definitive functional differences within *C. jirovecii*.

Searches against the Transporter Classification Database identified 644 genes associated with transport functions. Searches against PHI-base and the Database of Fungal Virulence Factors yielded 911 and 205 genes, respectively, encoding homologs linked to host interaction or potential pathogenicity-related functions. These results indicate that H0426_7 contains a broad set of genes associated with nutrient acquisition, stress response, and host-interaction-related homologs, although these homology-based annotations should not be interpreted as direct evidence of pathogenicity.

### 3.6. Average Nucleotide Identity Analysis

ANI analysis was performed to assess the genomic relatedness between strain H0426_7 and 14 publicly available *Cutaneotrichosporon* genomes, including two previously deposited datasets labeled as *C. jirovecii* (ERR2799408 and ERR3040836). Pairwise ANI values among all strains are summarized in Table S4.

**Figure 2:**
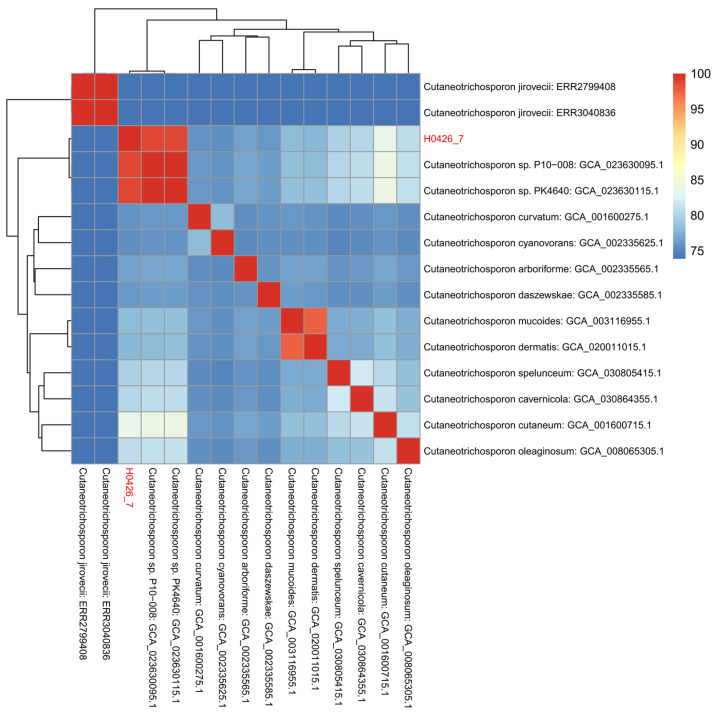
Hierarchical clustering based on average nucleotide identity distances among strain H0426_7 and representative *Cutaneotrichosporon* genomes. Heatmap colors represent pairwise ANI values, with red indicating high identity and blue indicating low identity. The dendrogram shows clustering based on ANI distances. Strain H0426_7 is highlighted in red and clusters closely with the unclassified strains P10-008 and PK4640.

Strain H0426_7 shared ANI values of 75.53–83.71% with recognized *Cutaneotrichosporon* species other than the two *C. jirovecii*-labeled datasets, all of which fall well below the commonly used 95% genome-level reference threshold for species delineation. In contrast, H0426_7 exhibited ANI values of 98.84% and 98.83% with two unclassified *Cutaneotrichosporon* strains, P10-008 and PK4640, respectively, indicating a very close genomic relationship among these three strains. The ANI between P10-008 and PK4640 was 99.96%, indicating an extremely close genomic relationship. Hierarchical clustering based on ANI distances showed that H0426_7, P10-008, and PK4640 formed a distinct cluster separated from all recognized *Cutaneotrichosporon* species included in the analysis, including *C. cutaneum*, *C. oleaginosum*, *C. dermatis*, and *C. mucoides* (Figure 2). The broad interspecific ANI range also indicated substantial genomic divergence within this basidiomycetous lineage.

Notably, the two publicly available datasets labeled as *C. jirovecii* (ERR2799408 and ERR3040836) showed unexpectedly low ANI values with H0426_7, 73.87% and 73.88%, respectively. These values were comparable to those observed between H0426_7 and more distantly related *Cutaneotrichosporon* species and were discordant with the ITS-based phylogenetic analysis, which placed H0426_7 within a *C. jirovecii*-related clade (see Section 3.7). To assess the possible cause of this discrepancy, we further examined the read-level taxonomic composition of ERR2799408 and ERR3040836 reported in the NCBI SRA. Reads assigned to *C. jirovecii* accounted for only 4.72% and 6.31% of the two datasets, respectively, whereas more than 90% of reads were unclassified or associated with non-target organisms. Such low target-read proportions are insufficient for reliable *de novo* assembly of a fungal genome and may lead to highly fragmented assemblies, contaminant-derived contigs, chimeric sequences, and apparent gene loss.

Therefore, the low ANI values between H0426_7 and the two *C. jirovecii*-labeled datasets should be interpreted cautiously and were not used as decisive evidence for species-level separation from authentic *C. jirovecii*. Instead, the most robust signal from the ANI analysis was the high genome-wide similarity among H0426_7, P10-008, and PK4640, suggesting that they represent a closely related genomic lineage within the *C. jirovecii*-related group.

### 3.7. Phylogenetic Analysis Based on ITS Sequences

**Figure 3:**
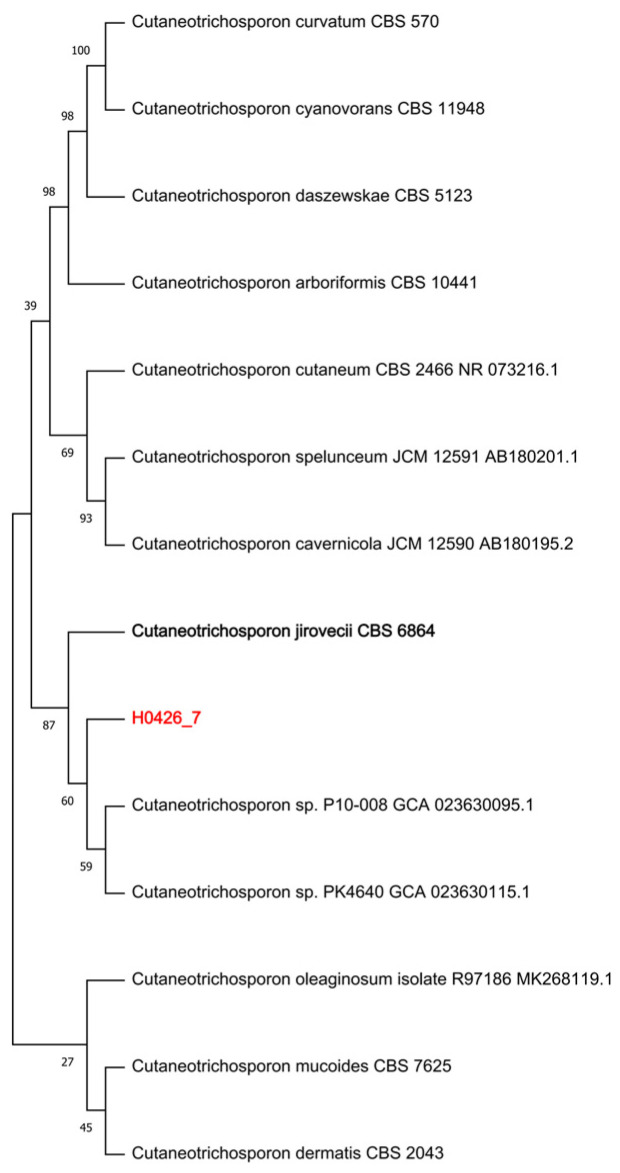
Neighbor-joining phylogenetic tree based on ITS sequences showing the phylogenetic position of strain H0426_7 within the genus *Cutaneotrichosporon*. The tree was constructed using the neighbor-joining method with 1000 bootstrap replicates. Bootstrap values greater than 50% are shown at branch nodes. Evolutionary distances were computed using the Maximum Composite Likelihood method. Strain H0426_7 is highlighted in red, and the type strain *C. jirovecii* CBS 6864 is indicated in bold.

The ITS-based neighbor-joining tree (Figure 3) placed strain H0426_7 within the *C. jirovecii*-related clade, together with the type strain *C. jirovecii* CBS 6864 and two genome-derived unclassified strains, *Cutaneotrichosporon* sp. P10-008 and PK4640. H0426_7 clustered adjacent to the type strain CBS 6864 with bootstrap support of 87%, indicating a well-supported ITS-based phylogenetic affinity between H0426_7 and type-strain material of *C. jirovecii*. The broader *C. jirovecii*-related clade was clearly separated from other *Cutaneotrichosporon* species, including *C. cutaneum*, *C. dermatis*, *C. mucoides*, *C. oleaginosum*, *C. spelunceum*, and *C. cavernicola*.

However, the internal topology within this clade was only weakly to moderately supported beyond the H0426_7/CBS 6864 relationship. The grouping of P10-008 and PK4640 was supported by a bootstrap value of 59%, indicating limited resolution for some internal relationships among the closely related genome-derived strains. These values indicate that ITS sequences are useful for placing H0426_7 within the *C. jirovecii*-related lineage but have limited resolution for clarifying all internal relationships and species boundaries within this group. This interpretation is also consistent with the ANI analysis, in which H0426_7 showed particularly high genome-wide similarity to P10-008 and PK4640.

Taken together, the ITS phylogeny supports the close relationship of H0426_7 to *C. jirovecii* type-strain material, whereas the ANI analysis highlights its strong genomic affinity with the two unclassified strains P10-008 and PK4640. Because a high-quality genome assembly of the *C. jirovecii* type strain CBS 6864 is currently unavailable for direct ANI comparison, the precise taxonomic boundary between H0426_7, the P10-008/PK4640 lineage, and authentic *C. jirovecii* remains unresolved. Accordingly, H0426_7 is best described as a *C. jirovecii*-related *Cutaneotrichosporon* isolate rather than being assigned unequivocally to *C. jirovecii* solely on the basis of ITS similarity.

## 4. Discussion

This study presents a draft genome sequence and comparative genomic analysis of H0426_7, a *Cutaneotrichosporon jirovecii*-related yeast recovered from a human fecal sample. The main contribution of this work is the integration of type-strain-oriented ITS comparison, ITS phylogenetic analysis, genome-wide ANI, read-level assessment of public *C. jirovecii*-labeled datasets, and comparative functional annotation into a single interpretive framework. This approach addresses a practical problem in fungal taxonomy and clinical microbiology: available genomic resources for *C. jirovecii* and related *Cutaneotrichosporon* lineages are limited, and some public datasets labeled as *C. jirovecii* are not suitable as reliable species-level genomic references. By placing H0426_7 in relation to the type strain CBS 6864 and to the feces-derived strains P10-008 and PK4640, this study provides a more precise framework for interpreting *C. jirovecii*-like isolates recovered from human-associated samples.

### 4.1. Taxonomic Placement and Genomic Divergence

The species-affinity analysis of H0426_7 illustrates both the utility and the limitation of ribosomal markers. Type-material-restricted BLAST analysis showed that the ITS sequence of H0426_7 closely matched *C. jirovecii* type-strain material, including CBS 6864 and its equivalent deposits. The ITS-based neighbor-joining tree further placed H0426_7 adjacent to CBS 6864 with bootstrap support of 87%, supporting its close affinity to the *C. jirovecii* type-strain lineage. However, ITS alone did not fully resolve all internal relationships within the broader *C. jirovecii*-related clade, consistent with the known limitations of single ribosomal markers in closely related basidiomycetous yeasts. The IGS1 sequence was examined but was not informative for type-strain-level assignment under current database coverage. Therefore, our taxonomic interpretation relied on the combined evidence from ITS phylogeny and genome-wide comparison rather than on a single marker.

Genome-wide ANI analysis provided an additional layer of resolution. H0426_7 showed high ANI values with *Cutaneotrichosporon* sp. P10-008 and PK4640, exceeding 98.8%, and the three strains formed a coherent genomic cluster. Notably, P10-008 and PK4640 were both derived from fecal samples of healthy human donors according to public BioSample metadata, suggesting that H0426_7 belongs to a small feces-associated genomic cluster rather than representing an isolated genome-level observation. However, the precise taxonomic boundary between this lineage and type-strain-defined *C. jirovecii* remains unresolved because a high-quality genome assembly of the *C. jirovecii* type strain CBS 6864 is not currently available for direct ANI comparison. Two public datasets labeled as *C. jirovecii* (ERR2799408 and ERR3040836) showed low ANI values with H0426_7 (73.87% and 73.88%). Read-level taxonomic profiling indicated extremely low proportions of target fungal reads (4.72% and 6.31%) and a high proportion of unclassified or non-target reads. These datasets were therefore not treated as reliable references for species-level ANI interpretation. On this basis, H0426_7 is best described conservatively as a *C. jirovecii*-related *Cutaneotrichosporon* isolate.

### 4.2. Genome Quality and Filtering Strategy

The draft genome of H0426_7 comprises 1974 contigs with an N50 of 30.3 kb and a BUSCO completeness of 80.0%. These metrics indicate a moderately fragmented assembly, which should be considered when interpreting gene-content analyses. The initial assembly before post-assembly filtering contained 86.4% complete BUSCOs. The reduction to 80.0% reflected a stringent filtering strategy that included reference-guided exclusion of contigs without significant *Cutaneotrichosporon* matches, CD-HIT redundancy removal, and length filtering. This strategy prioritized a conservative, non-redundant gene set for downstream analyses, although it also reduced apparent BUSCO completeness. The elevated proportion of duplicated BUSCOs (43.0%) may reflect biological features of basidiomycetous yeasts, such as gene duplication, structural variation, and unresolved allelic variation, combined with technical limitations of short-read assembly in repetitive or high-GC regions. The overall GC content of H0426_7 (63.76%) was comparable to values reported for other members of the genus *Cutaneotrichosporon*, suggesting that the observed assembly characteristics are broadly consistent with this lineage. Long-read sequencing will be required to obtain a more contiguous assembly and resolve complex genomic regions.

### 4.3. Clinical and Ecological Context

The isolate was recovered from a fecal specimen of a *C. difficile* toxin-positive patient, and it was the only yeast-like colony morphotype observed on the primary SDA plate, with a semi-quantitative growth density recorded as “+”. This finding confirms recovery of the organism from the fecal sample but does not establish stable colonization, pathogenicity, or any causal role in CDI. The CDI status of the patient should therefore be interpreted as a background of intestinal dysbiosis in which uncommon or low-abundance fungi may become detectable. This interpretation is consistent with the broader concept that antibiotic-associated disruption of the gut ecosystem may alter fungal community structure [14,15], but the present study does not test that hypothesis directly.

The potential under-recognition of *C. jirovecii*-related yeasts in clinical microbiology is also plausible because routine phenotypic and MALDI-TOF MS approaches may fail to resolve uncommon *Trichosporon*/*Cutaneotrichosporon* isolates to the species level when reference databases are incomplete. Published reports of *C. jirovecii* or closely related taxa in oral, urinary, cutaneous, respiratory, and animal-associated contexts further suggest that this species may occur across a broader range of host-associated niches than currently appreciated [2,3,7,8,10,11,12]. Notably, the close genomic relationship between H0426_7 and the feces-derived strains P10-008 and PK4640 suggests that recovery of members of this lineage from human-associated gastrointestinal samples may not be an isolated observation. High-quality genomic resources are therefore needed not only for taxonomy but also for improving species-level identification in diagnostic workflows.

### 4.4. Functional Features and Metabolic Potential

CAZyme annotation identified 285 family assignments representing 278 non-redundant predicted proteins in H0426_7, placing it within the upper range of the compared *Cutaneotrichosporon* genomes. Its CAZyme repertoire was closer in size to those of P10-008 and PK4640, which contained 351 and 353 CAZyme family assignments, respectively, than to several recognized species with smaller repertoires. At the family level, H0426_7 and the two closely related feces-derived strains shared relatively high counts of GH5 and GH31 assignments. GH5 enzymes include activities related to the degradation of cellulose, hemicellulose, and other plant cell wall polysaccharides, whereas GH31 includes α-glucosidases involved in the hydrolysis of starch-derived α-linked glucans. These observations suggest that the H0426_7/P10-008/PK4640 lineage may possess a relatively expanded carbohydrate-utilization repertoire. Given the limited number of available strains and the draft nature of the assemblies, this pattern should be viewed as hypothesis-generating rather than as evidence of gut adaptation.

The recurrent clustering of H0426_7 with the feces-derived strains P10-008 and PK4640 is noteworthy from an ecological perspective. Although recovery from fecal material alone does not establish stable gastrointestinal colonization, independent recovery of three closely related genomes from human fecal samples suggests that this lineage may be repeatedly associated with the human gastrointestinal environment. When considered together with the relatively expanded GH5 and GH31 repertoires, these observations raise the possibility that members of this lineage possess metabolic capabilities compatible with survival in carbohydrate-rich host-associated habitats. Additional sampling and longitudinal studies will be required to determine whether these organisms represent transient passengers, opportunistic colonizers, or persistent members of the human gut mycobiome.

In addition to CAZyme repertoires, H0426_7 contained numerous transporter-associated genes and homologs identified through PHI-base and fungal virulence-related databases. Other annotation results also support metabolic versatility rather than overt pathogenic specialization. Transporter-associated genes, oxidoreductase-related functions, and homologs found in host-interaction or virulence-related databases were detected in H0426_7. For environmental and opportunistic yeasts, such homologs often reflect conserved eukaryotic survival mechanisms, stress response, and nutrient acquisition rather than dedicated virulence programs. Their presence should not be interpreted as evidence of pathogenicity; instead, they provide candidate features for future phenotypic assays.

### 4.5. Antifungal Susceptibility

The antifungal susceptibility profile of H0426_7 was interpreted descriptively because no species-specific breakpoints or epidemiological cutoff values are available for *Cutaneotrichosporon*. Using the Sensititre YeastOne colorimetric microdilution panel, H0426_7 showed low MIC values for triazoles, amphotericin B, echinocandins, and flucytosine. Similar MIC patterns have been reported in other members of Trichosporonaceae. However, because interpretive criteria are not established for this genus and because a commercial colorimetric method was used, these MICs should not be translated into categorical susceptibility or resistance conclusions. Larger isolate collections and standardized testing will be required to evaluate whether consistent susceptibility patterns exist in this lineage.

The antifungal susceptibility profile observed in H0426_7 was generally consistent with the limited data currently available for Trichosporonaceae and related basidiomycetous yeasts. Although definitive genotype–phenotype relationships cannot be inferred from a single draft genome, accumulation of genomic and phenotypic datasets from additional isolates may ultimately facilitate identification of lineage-specific determinants associated with antifungal susceptibility variation.

Future comparative analyses incorporating larger collections, higher-quality genome assemblies, and additional genomic features such as transcription factors, protein kinases, and transposable elements may provide further insight into antifungal susceptibility variation within this lineage.

### 4.6. Limitations

Several limitations should be acknowledged. First, this study is based on a single clinical fecal isolate and does not include prevalence analysis, longitudinal follow-up, or matched healthy controls. Therefore, it cannot determine whether H0426_7 represented transient passage, short-term colonization, or a stable component of the gut mycobiome. Second, the genome remains a short-read draft assembly with moderate BUSCO completeness and substantial fragmentation. Third, functional interpretation was based on homology-based annotation and requires experimental validation. Fourth, available public *C. jirovecii*-labeled genome datasets were not suitable as definitive references because of low target-read proportions and possible contamination. Fifth, the lack of a high-quality genome assembly from the *C. jirovecii* type strain CBS 6864 remains a major obstacle to formal taxonomic resolution. Sixth, although CAZyme repertoires provided a useful framework for comparative functional analysis, other genomic features such as transcription factors, protein kinases, DNA-binding proteins, transposable elements, and codon usage patterns may also contribute to ecological adaptation and evolutionary diversification within Trichosporonaceae. Although these genomic features may provide additional biological insights, we focused on CAZyme repertoires because they could be robustly annotated and directly compared across all currently available *Cutaneotrichosporon* genomes using a unified analytical pipeline. Comprehensive characterization of these features is currently constrained by the fragmented nature of the available assembly and the limited number of closely related genomes.

## 5. Conclusions

In summary, this study provides a draft genome and comparative genomic analysis of a *C. jirovecii*-related *Cutaneotrichosporon* isolate recovered from a human fecal sample. ITS analysis supports its close relationship to *C. jirovecii* type-strain material, whereas ANI analysis links it strongly to two feces-derived unclassified *Cutaneotrichosporon* strains. Comparative functional annotation identifies an expanded CAZyme repertoire, particularly involving selected carbohydrate-degrading families, as a candidate feature of this lineage. These findings expand genomic resources for underrepresented basidiomycetous yeasts, refine the placement of H0426_7 within the *C. jirovecii*-related group, and provide a foundation for future studies on the taxonomy, ecology, antifungal susceptibility, and functional biology of feces-associated *Cutaneotrichosporon* lineages.

## Figures and Tables

**Table 1:** Clinical characteristics and antifungal susceptibility profile of strain H0426_7.

Clinical Feature	Patient
Age (years)	69
Gender	Male
Department	Department of Gastroenterology
Reason for hospital admission	Diarrhea
*Clostridioides difficile* toxin	Positive
Stool consistency	Soft
Stool color	Yellow
Fungal growth on SDA	“+”
Antifungal agent	MIC (µg/mL)
Anidulafungin	0.25
Micafungin	0.5
Caspofungin	0.25
5-Flucytosine	2
Posaconazole	0.06
Voriconazole	0.015
Itraconazole	0.06
Fluconazole	0.5
Amphotericin B	0.25

**Table 2:** Genome sequencing and assembly statistics of the strain H0426_7.

Item	Value
Sequencing platform	Illumina paired-end
Total clean reads	13,884,374
Total clean base pairs	2.09 Gb
Average sequencing depth	54×
Total assembled genome size	38,658,432 bp (38.66 Mb)
Number of contigs	1974
GC content	63.76%
Longest contig	201,186 bp
Contig N50	30,278 bp
Complete BUSCOs	80.0%
Single-copy BUSCOs	37.0%
Duplicated BUSCOs	43.0%
Fragmented BUSCOs	1.7%
Missing BUSCOs	18.3%

**Table 3:** Distribution of CAZyme family assignments across *Cutaneotrichosporon* strains. CBM, carbohydrate-binding module; CE, carbohydrate esterase; GH, glycoside hydrolase; GT, glycosyltransferase; PL, polysaccharide lyase; AA, auxiliary activity. H0426_7 is highlighted in bold. For all strains, class counts correspond to CAZyme family assignments. For H0426_7, 285 assignments represented 278 non-redundant predicted proteins because seven predicted proteins were assigned to more than one CAZy family or class. The two public datasets labeled as *C. jirovecii* were included only as exploratory references because of read-level quality concerns discussed in Section 3.6.

Strain	CBM	CE	GH	GT	PL	AA	Total
*Cutaneotrichosporon jirovecii*: ERR2799408	8	2	54	35	1	7	107
*Cutaneotrichosporon jirovecii*: ERR3040836	9	2	56	36	1	7	111
**H0426_7**	**28**	**6**	**138**	**79**	**4**	**30**	**285**
*Cutaneotrichosporon* sp. P10-008: GCA_023630095.1	46	6	163	93	4	39	351
*Cutaneotrichosporon* sp. PK4640: GCA_023630115.1	45	6	162	97	5	38	353
*Cutaneotrichosporon curvatum*: GCA_001600275.1	18	1	68	45	3	16	151
*Cutaneotrichosporon cutaneum*: GCA_001600715.1	24	2	82	50	3	19	180
*Cutaneotrichosporon arboriforme*: GCA_002335565.1	15	2	63	41	3	18	142
*Cutaneotrichosporon daszewskae*: GCA_002335585.1	19	3	95	49	4	20	190
*Cutaneotrichosporon cyanovorans:* GCA_002335625.1	17	1	69	44	3	17	151
*Cutaneotrichosporon mucoides*: GCA_003116955.1	40	3	170	93	8	35	349
*Cutaneotrichosporon oleaginosum*: GCA_008065305.1	18	2	62	47	3	16	148
*Cutaneotrichosporon dermatis*: GCA_020011015.1	36	4	154	89	7	30	320
*Cutaneotrichosporon spelunceum*: GCA_030805415.1	18	1	67	44	2	16	148
*Cutaneotrichosporon cavernicola*: GCA_030864355.1	15	1	81	46	3	18	164

## Data Availability

The draft genome sequence of the strain H0426_7 has been deposited in the NCBI GenBank database under accession number JBVFRA000000000. Raw sequencing reads generated in this study are available in the NCBI Sequence Read Archive under accession number SRR37291376. The associated BioProject and BioSample records are available under accession numbers PRJNA1420265 and SAMN55214668, respectively.

## Supplementary Materials

The following supporting information can be downloaded at: https://www.mdpi.com/article/10.3390/jof12060450/s1. Supplementary Table S1. BLASTn comparison of ITS and IGS1 sequences of strain H0426_7 against reference/type-material records. Supplementary Table S2. Summary of functional annotation results for strain H0426_7. Supplementary Table S3. Family-level CAZyme assignments, including GH5 and GH31, across H0426_7 and representative *Cutaneotrichosporon* genomes. Supplementary Table S4. Pairwise average nucleotide identity values among strain H0426_7 and representative *Cutaneotrichosporon* genomes. Supplementary Table S5. *Cutaneotrichosporon* reference genomes used for BLASTn-based contig filtering.
